# Synthesis of biochar-supported sulfidated nanoscale zero-valent iron and its application as a persulfate activator for remediation of crude oil-contaminated soil

**DOI:** 10.3389/fmicb.2026.1783533

**Published:** 2026-04-14

**Authors:** Jin Zheng, Yong Zhai, Chunyang Gao, Mengmeng Niu, Jufeng Li, Hongkun Chen, Jun Xu, Wenwen Wang, Qian Wu, Hongyun Zhao, Ziqiu Nie, Xianyuan Du

**Affiliations:** 1 State Key Laboratory of Petroleum Pollution Control, Beijing, China; 2 CNPC Research Institute of Safety and Environmental Technology, Beijing, China; 3 China Kunlun Contracting and Engineering Co., Ltd., Jilin Branch, Jilin, China; 4 College of Materials Science and Engineering, Shenyang Jianzhu University, Shenyang, China; 5 College of Water Sciences, Beijing Normal University, Beijing, China

**Keywords:** biochar-supported sulfidated nanoscale zero-valent iron, crude oil-contaminated soil, microbial community, sodium persulfate, soil toxicity

## Abstract

Petroleum contamination poses a serious threat to soil ecosystems and microbial communities. Persulfate (PS)-based advanced oxidation has shown promising remediation potential; however, conventional nanoscale zero-valent iron (nZVI) suffers from surface passivation, aggregation, and limited stability in complex soil systems. To address these challenges, a biochar-supported sulfidated nanoscale zero-valent iron composite (S-nZVI@BC) was synthesized via liquid-phase reduction to enhance PS activation efficiency and improve remediation performance in crude oil-contaminated soil. Material characterization confirmed the successful loading of sulfidated nZVI onto biochar with good dispersion. At a biochar loading rate of 25%, S/Fe molar ratio of 1:10, and activated carbon particle size of 0.83 mm, the S-nZVI@BC/PS system achieved the best crude oil degradation effect. Moreover, a maximum degradation efficiency of 90.14% was achieved by employing PS and S-nZVI@BC dosages of 2 and 10%, respectively, at a water-soil ratio of 1:1. SO_4_^–^∙ and ∙OH are the primary species responsible for crude oil degradation, and the contribution of SO_4_^–^∙ was greater than that of ∙OH. After the reaction, significant reductions were observed in saturated hydrocarbons, aromatic hydrocarbons, resins, and asphaltenes. Meanwhile, The soil remediated by S-nZVI@BC/PS altered the microbial community structure and increased the relative abundance of petroleum degradation-related microorganisms. The germination rate of *Elymus dahuricus* increased markedly, approaching that of uncontaminated soil. These findings highlight the potential of the S-nZVI@BC/PS system as an efficient and sustainable strategy for the remediation of crude oil-contaminated soils.

## 1 Introduction

Petroleum leakage is frequently observed during extraction, production, transportation, and storage due to technical limitations, equipment failures, and poor management practices (Mekonnen et al., 2023). For instance, over 600,000 leaking underground storage tank sites have been documented in the United States alone (US EPA, 2022). Furthermore, regions such as the Niger Delta have experienced thousands of spill incidents over several decades, resulting in persistent hydrocarbon contamination of soils and aquifers (United Nations Environment Programme [UNEP], 2011; Nriagu et al., 2016). Petroleum pollutants are complex multicomponent mixtures which are highly resistant to degradation and persist in soil for extended periods (Rahmawati et al., 2023). Therefore, efficient and practical technologies for removing crude oil contaminants from soil are urgently needed.

Common remediation approaches include incineration (Huang et al., 2024), thermal desorption (Chen et al., 2024), soil vapor extraction (Liu et al., 2024), electrokinetic remediation (Fu et al., 2017), biodegradation (Deng et al., 2020), and advanced oxidation processes (Ma et al., 2025; Zeng et al., 2025; Liu et al., 2023). Among these methods, advanced oxidation has been widely applied because of its high removal efficiency and short treatment time. Common oxidants include hydrogen peroxide (Wang J. et al., 2023), potassium permanganate (Fu et al., 2025), and PS (Liu et al., 2023). PS can generate SO_4_^–^∙ (*E_*h*_* = 2.5–3.1 V), which possess a higher redox potential than ∙OH (*E_*h*_* = 1.7–2.7 V). Various half-lives (30–40⋅μs) have been reported (Xia et al., 2020). The system also remains effective over a broad pH range (Anh and Young, 2022). Chen L. et al. (2019) reported that the PS/nano zero-valent iron (nZVI) system exhibited a significantly higher degradation performance for PAHs than peroxymonosulfate/nZVI and hydrogen peroxide/nZVI systems. Quenching experiments confirmed that SO_4_^–^∙ was the dominant reactive species. Recently, nZVI has attracted significant attention due to its high reactivity, ability to gradually release Fe^2+^, and low toxicity. Under acidic conditions, nZVI can be directly oxidized to Fe^2+^ to activate PS. PS can also be activated through electron transfer from nZVI, generating SO_4_^–^∙ Fe^2+^ can be continuously released through simultaneous reactions of nZVI with H_2_O, O_2_, and PS. However, the formation of iron oxides and hydroxides during the reaction leads to surface passivation of nZVI, which inhibits Fe^2+^ release (Yao et al., 2020).

The tendency of nZVI to aggregate and oxidize further limits its practical applications (Zhou et al., 2023a). To overcome the limitations associated with surface passivation and particle aggregation, various supporting materials have been introduced to improve the stability and dispersion of nZVI. Among them, biochar has attracted considerable attention due to its porous structure, high specific surface area, and abundant surface functional groups. As a carbonaceous support, biochar can effectively disperse nZVI nanoparticles, reduce aggregation, and enhance electron transfer processes, thereby promoting Fe^2+^ regeneration and PS activation efficiency. Meanwhile, the incorporation of sulfur species onto the surface of nZVI can suppress hydrogen evolution side reactions and improve pollutant selectivity, electron transfer efficiency, and particle stability (Li et al., 2021). Field-scale injection experiments have demonstrated that sulfidated nZVI (S-nZVI) exhibits good mobility and distribution in aquifers (Garcia et al., 2020), whereas FeS-modified composites have achieved highly efficient trichloroethene degradation through PS activation (Sun et al., 2020; Dong et al., 2018). Moreover, S-nZVI-based PS systems have shown strong efficacy in degrading various organic contaminants (Yu et al., 2022; Guo et al., 2020) and arsenic removal (Singh et al., 2021). These findings confirm that S-nZVI can activate PS to generate reactive oxygen species such as sulfate radicals (SO_4_^–^∙) and hydroxyl radicals (∙OH), thereby enabling rapid and efficient pollutant degradation (Rayaroth et al., 2020; Fan et al., 2022).

Therefore, the S-nZVI@BC composite was synthesized by loading S-nZVI onto biochar (BC). Specifically, this study included the following: (1) The synthesis conditions for S-nZVI@BC were optimized using the liquid-phase reduction method. (2) The effects of key factors, including PS dosage, composite dosage, water-to-soil ratio, pH, inorganic ions, PS addition frequency, and different catalytic systems, on crude oil degradation efficiency were evaluated. (3) A degradation mechanism was proposed based on quenching experiments and structural characterization. (4) The effect of the S-nZVI@BC/PS system on soil functionality was assessed. This study provides a more comprehensive framework for understanding oxidation-driven soil remediation, thereby advancing sustainable remediation technologies for complex petroleum-contaminated environments.

## 2 Materials and methods

### 2.1 Materials

All analytical-grade chemical reagents were purchased from commercial sources and used without further purification. Detailed information can be found in Supplementary Test S1.

### 2.2 Synthesis of S-nZVI@BC composite material

Synthesis of S-nZVI@BC using liquid-phase reduction: Coconut shells were oven-dried at 105°C, crushed, and carbonized in a tubular furnace at 450–500°C for 3 h. The resulting BC was activated with steam and flue gas at 850–950°C under 1 MPa using gas-phase activation. The BC was then acid-washed to improve surface adsorption and remove impurities, rinsed with deionized water, dried, and sieved for subsequent use.

Synthesis of S-nZVI@BC in a three-necked flask (Supplementary Figure S1): The obtained BC was used as a carrier to fabricate S-nZVI@BC. First, 3.2 g FeSO_4_.7H_2_O, 0.1 g Na_2_S_2_O_4_, and 2.56 g BC were added to a mixture of anhydrous ethanol and deionized water (volume ratio: 3:7). Second, the mixture was placed in a three-necked flask and ultrasonicated for 15 min. Third, 50 mL of 0.94 M NaBH_4_ was added dropwise under continuous stirring, and the drop process lasted approximately 20 min. Finally, the resulting precipitate was separated using a magnet and sequentially washed with deionized water and anhydrous ethanol to obtain S-nZVI@BC via vacuum drying. All deionized water and anhydrous ethanol used in the preparation of S-nZVI@BC were treated with a nitrogen purge to minimize the effect of oxygen. The entire preparation process of the composite material was performed in a nitrogen atmosphere. The nZVI, S-nZVI, and nZVI@BC materials were fabricated following the procedure similar to that of S-nZVI@BC, except whether FeSO_4_.7H_2_O and BC were added.

To optimize the design of the composite material, the effects of carbon loading, S/Fe molar ratio, and BC particle size on the degradation properties of the composite materials were systematically analyzed. The carbon-loading ratios were set to 5, 10, 15, 25, and 35%. Five S/Fe molar ratios, namely 1:3, 1:5, 1:10, 1:50, and 1:100, were used in this study, with BC carriers of 850–425, 425–180, 180–150, and 125–106 μm, respectively.

### 2.3 Degradation experiment of crude oil-contaminated soil

Crude oil-contaminated soil was collected from sites near an oil field in northern China. In the laboratory, the contaminated soil was air-dried and sieved to < 595 μm. Subsequently, the soil was refrigerated at −20°C. The basic indicators of the contaminated soil are listed in Supplementary Table S1. Two grams of soil was initially added to a conical flask. Then, a certain volume of PS (0.84 M) and a given mass of composite materials were added. The soil–water mass ratio was maintained at a given level by adding deionized water, and the mixture was sufficiently stirred to ensure that the materials were fully in contact with the contaminated soil. Degradation temperature was set as 25 ± 1°C and reaction time was 48 h. The mixture was stirred regularly during the degradation. The mixture was stirred regularly during the degradation. The total petroleum content was measured in accordance with the Chinese standard method HJ 1051–2019 (Infrared Spectrophotometry). The concentrations of n-alkanes (C10-C40) were analyzed following HJ 1021–2019 using gas chromatography.

### 2.4 Free radical detection experiment

Electron paramagnetic resonance (EMPlus-6/1/p/L, Bruker, Bremen, Germany) was employed to identify the reactive free radicals in the degradation system, with DMPO and TEMP as the spin-trapping reagents. Subsequently, the contributions of various free radicals to the crude oil removal efficiency were analyzed via quenching experiments. Four quenching agents, methanol (MeOH), tert-butyl alcohol (TBA), p-benzoquinone (PBQ), and potassium iodide (KI) were used to inhabit the activity of SO_4_^–^∙, ∙OH, ^1^O_2_, and surface reaction, respectively (Liu et al., 2023; Zheng et al., 2024).

### 2.5 Wheat germination test

An ecotoxicological assessment of the polluted soil before and after remediation was conducted using wheat as a bioindicator. First, plump and uniform wheat seeds were washed three times with anhydrous ethanol. The seeds were then soaked in deionized water for 2–3 h, and dried using filter paper. Finally, the seeds were planted in a Petri dish containing 100 g of soil. The culture temperature was 25°C and soil water content was approximately 60%. After 5 days, seeds with root lengths > 5 mm were counted, and the sprouting rate was determined.

### 2.6 Analytical methods

Field-emission scanning electron microscopy with energy dispersive spectroscopy (SEM-EDS; S-4800, Hitachi, Tokyo, Japan) was used to observe the surface morphology of the materials. The Fourier transform infrared (FT-IR) spectra in the 4,000–400 cm^–1^ region were obtained on a FT-IR spectrometer (United States Nicolet iS10). The crystal structure of materials was recorded using powder X-ray diffractometry (XRD; Cu-Kα radiation source, Ultima IV, Rigaku, Tokyo, Japan) in the 2θ range from 1° to 90°. The surface elemental compositions and chemical bonding status of materials were recorded using X-ray photoelectron spectroscopy (XPS; EscaLab 250Xi, United States) using an Al-Kα excitation source.

## 3 Results

### 3.1 Characterizations

**FIGURE 1:**
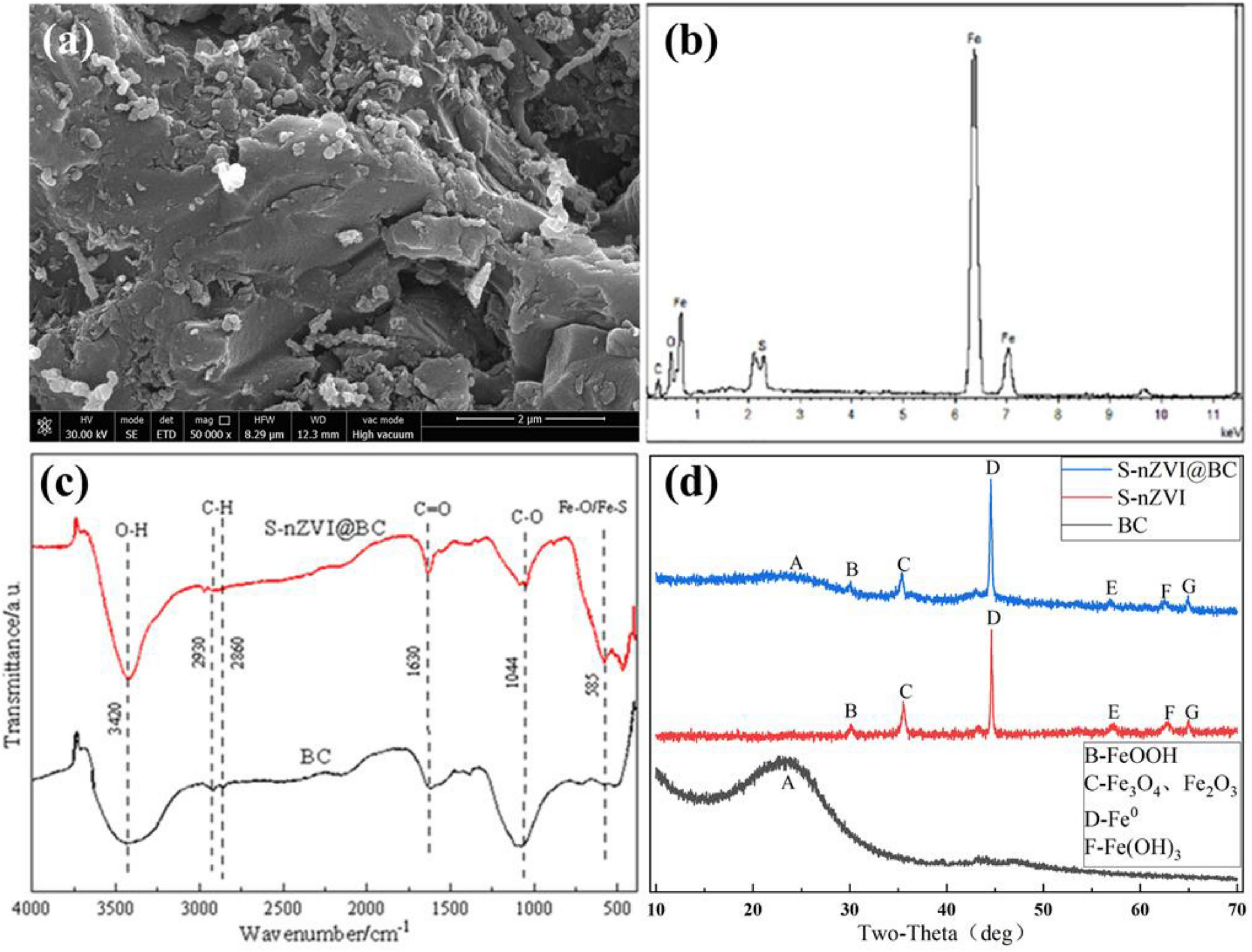
**(a)** SEM image and **(b)** EDS spectrum of S-nZVI@BC. **(c)** FT-IR and **(d)** XRD spectra.

To confirm the structure of the composite materials, SEM-EDS, FT-IR, XRD, and XPS analyses were performed. As shown in Supplementary Figure S2, smooth-surfaced nZVI is distributed in a spherical chain shape. After sulfurization, the S-nZVI surface becomes uneven and rough (Supplementary Figure S3), which can be attributed to the presence of FeS_*x*_ on the surfaces of the particles. Overall, nZVI and S-nZVI exhibited irregular and uncontrolled agglomeration. When lamellar BC was introduced, the agglomeration of the S-nZVI particles was significantly inhibited (Figure 1a). This increased the contact between S-nZVI and PS, thereby improving the ability of S-nZVI@BC to degrade crude oil. The EDS spectrum in Figure 1b indicates that four elements, namely iron, sulfur, carbon, and oxygen, are distributed on the S-nZVI@BC surface. The presence of sulfur implied that the sulfurization modification of S-nZVI@BC was successful, and the presence of oxygen suggested that the composite material was partly oxidized by air. The FT-IR spectra of the BC and the composite material are shown in Figure 1c. For S-nZVI@BC, the O-H, C = O, and C-O stretching vibrations are located at the adsorption peaks at 3,420, 1,630, and 1,044 cm^–1^, respectively. The peaks at 2,930 and 2,860 cm^–1^ are due to the symmetric and asymmetric stretching of C-H (Parikh et al., 2014). These results were also observed in the BC spectrum. Nevertheless, compared with BC, the appearance of a new peak at 585cm^–1^ indicates the formation of Fe-O or Fe-S bonds in the composite, providing clear evidence for the successful incorporation of S-nZVI (Losacco et al., 2022; Lehmann and Joseph, 2015).

Figure 1d presents the XRD spectra of BC, S-nZVI, and S-nZVI@BC. The observed diffraction peaks of S-nZVI@BC at 30.92°, 44.56°, and 62.21° are ascribed to the FeOOH, Fe^0^, and Fe(OH)_3_ crystal phases, respectively (Uhm et al., 2004). The high Fe^0^ diffraction peak intensity was mainly attributed to the high crystallinity and high content of zero-valent iron in the composite material. The peaks at 35.38° and 56.89° originated from Fe_3_O_4_ or Fe_2_O_3_, respectively, and the broad peak at 15–30° belongs to the amorphous graphite phase (Cao et al., 2022). Notably, the peak at 64.86° implied that a certain amount of FeS existed on the surface of S-nZVI@BC. These diffraction peaks were observed for all S-nZVI, except for the graphite phase. In the BC spectrogram, only a broad peak at 15–30° was found, and no diffraction peak was related to S-nZVI.

**FIGURE 2:**
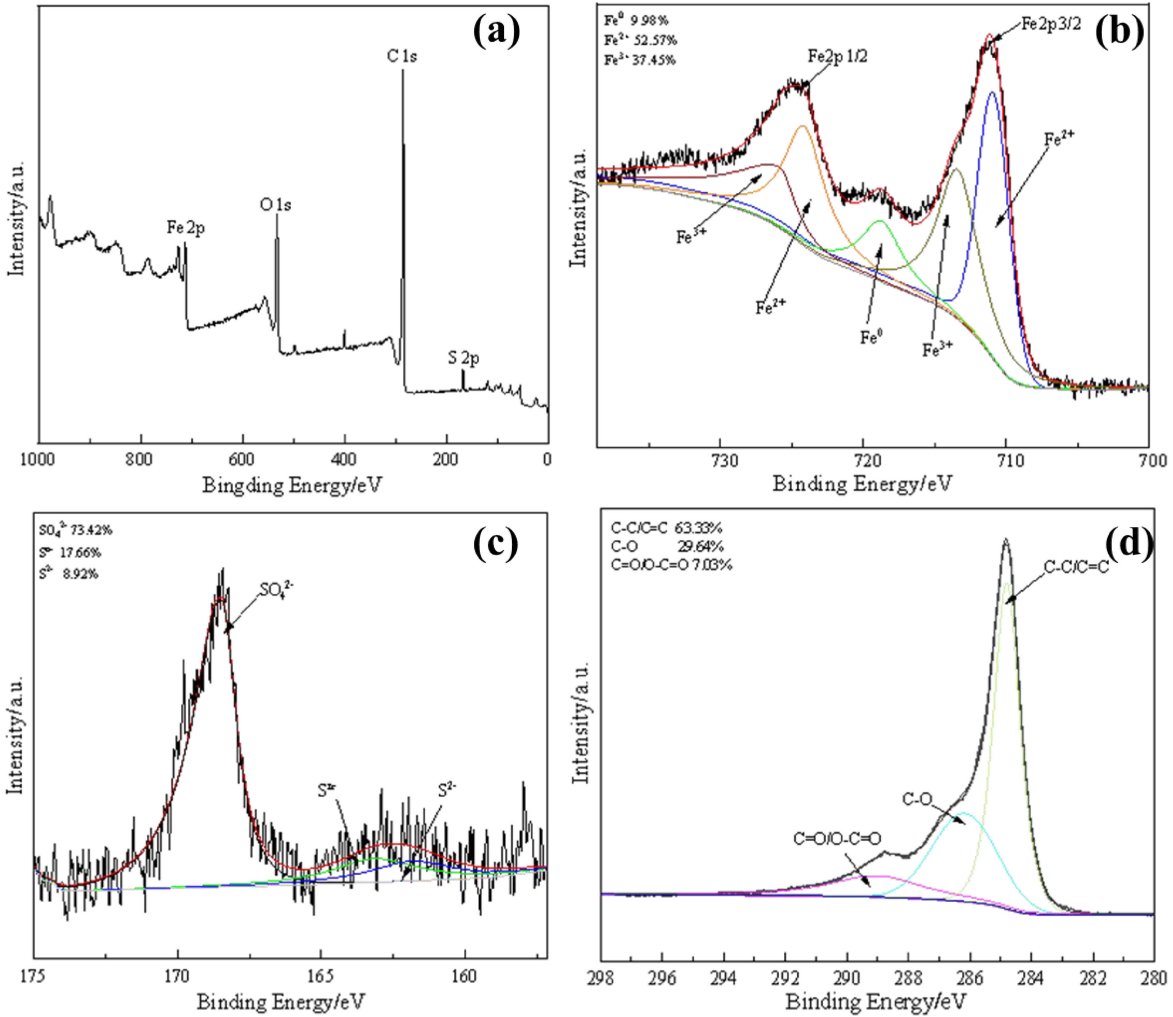
XPS spectra of S-nZVI@BC: **(a)** survey spectrum; fitting curves for **(b)** Fe2p, **(c)** S2p, and **(d)** C1s.

The XPS spectra were obtained to analyze the surface chemical bonding status of S-nZVI@BC. The survey XPS spectrum in Figure 2a indicates the existence of Fe2p, S2p, C1s, and O1s peaks on the surface of the composite material. The Fe2p spectrum in Figure 2b, shows obvious Fe2p_3/2_ and Fe2p_1/2_ peaks. This spectrum fits into five peaks: the peaks at 710.8 and 724.0 eV are ascribed to Fe^2+^, those at 713.9 and 725.5 eV are indexed to Fe^3+^, and that at 718.7 eV corresponds to Fe^0^. These results are also consistent with the multiform valence states of iron observed in the XRD analysis. In the S2p spectrum in Figure 2c, peaks at 161.0 eV, 163.1 eV, and 168.3 eV are associated with S^2–^, S^*n*–^, and SO_4_^2–^ species in S-nZVI@BC, respectively. S^2–^ can promote the cycle of Fe^3+^ and Fe^2+^ by releasing electrons, resulting in a high activation ability of the composite material. The C1s spectrum in Figure 2d can be fitted to three peaks corresponding to the C = C/C-C, C-O, and C = O/O-C = O bonds. These oxygen-containing functional groups act as active sites for PS activation. Moreover, they may act as significant anchor points that induce S-nZVI formation on the BC surface.

**FIGURE 3:**
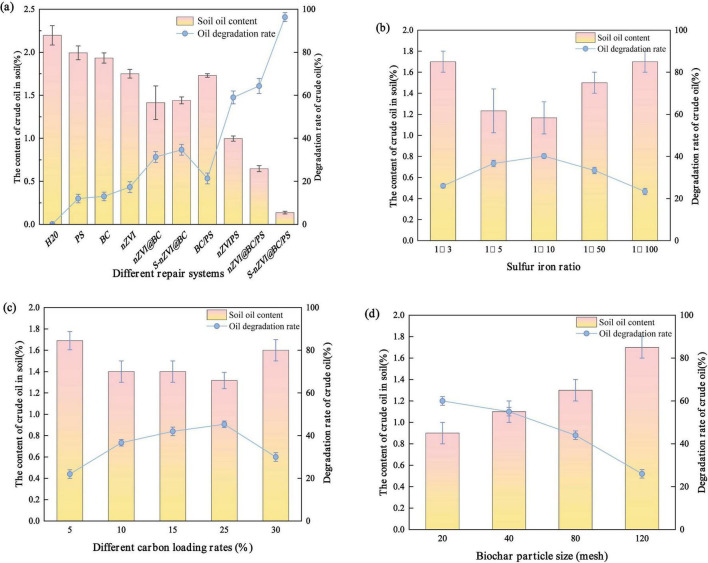
**(a)** Comparison of degradation effects of different remediation systems on crude oil-contaminated soil. Degradation efficiency of S-nZVI@BC on crude oil in soil: optimization of **(b)** S/Fe molar ratio, **(c)** carbon loading rate, and **(d)** BC particle size.

The S/Fe molar ratio indirectly influenced the crude oil degradation ability of the composite materials by affecting the cycle efficiency between Fe^3+^ and Fe^2+^. As shown in Figure 3b, as the S/Fe molar ratio increases, the crude oil degradation efficiency first increases and then decreases. Specifically, when the S/Fe molar ratios were 1:3, 1:5, 1:10, 1:50, and 1:100, the degradation efficiencies were 27.02, 38.52, 41.61, 33.50, and 22.60%, respectively. An increase in the S/Fe ratio facilitated the generation of additional FeS, which enhanced the surface roughness of S-nZVI@BC, and consequently increased its specific surface area. This increased the surface area of the material. Meanwhile, FeS deposits on nZVI can stabilize particles by wrapping individual nZVI particles and inhibiting their aggregation by steric hindrance. However, when FeS particles are present in excess, they can partially cover the active sites and weaken the activation ability toward the PS molecules. In addition, a higher S/Fe ratio can intensify the transformation from S-nZVI@BC to ferrous/iron (hydrogen) oxide, decreasing the degradation ability of the composite materials. The experimental results indicate that 1:10 is the optimal S/Fe molar ratio.

In addition to the S/Fe molar ratio, the effects of the carbon-loading rate on the crude oil-degradation efficiency of S-nZVI@BC were investigated. As shown in Figure 3c, when the carbon loading rate increases from 5 to 25%, the degradation efficiency increases from 22.6 to 41.17%. Increasing the carbon-loading rate of S-nZVI@BC improved the dispersion of S-nZVI particles on the BC surface, thereby providing more accessible reactive sites for PS activation. Furthermore, the oxygen-containing functional groups on the BC surface can activate PS to generate free radicals, and this activation ability is stronger at high carbon loading rates. However, when the carbon-loading rate was increased to 30%, the degradation rate of crude oil decreased by 11.5%. This is likely attributable to the high carbon loading. Excess S-nZVI blocked the pore structure of BC, reduced its adsorption capacity, and inhibited crude oil degradation by the S-nZVI@BC/PS system. To obtain optimal degradation efficiency, the carbon loading rate of S-nZVI@BC was set to 25%.

The degradation efficiency (Figure 3d) suggests that obviously lower removal ability is observed at smaller BC particle sizes, and the degradation efficiencies at 850–425, 425–180, 180–150, and 125–106 μm BC are 58.42, 50.12, 43.21, and 25.69%, respectively. Compared to small particles, large BC particles possess a good interpenetrating channel structure, which is suitable for depositing S-nZVI particles. These channels were sufficiently large for the diffusion of crude oil from the soil surface to the active sites of S-nZVI@BC. As the particle size decreased, the channel size of BC gradually decreased, resulting in poor deposition of S-nZVI on the BC surface and limited degradation ability of the composite material. In addition, the presence of narrow pores may have restricted the diffusion of crude oil within the S-nZVI@BC structure, further reducing its degradation efficiency. Based on these analyses, the optimal preparation conditions for S-nZVI@BC were determined to be an S/Fe molar ratio of 1:10, carbon loading rate of 25%, and BC particle size of 850–425 μm.

### 3.2 The different influencing factors of degradation properties

#### 3.2.1 Crude oil degradation by S-nZVI@BC/PS

In this study, 10 remediation systems were compared for their effectiveness in degrading crude oil in the soil (Figure 3a). The S-nZVI@BC system achieved degradation rates that were 18.03 and 4.71% higher than those of the nZVI and nZVI@BC systems, respectively. This improvement is attributed to the enhanced electron transfer efficiency and higher selectivity of S-nZVI@BC. The FeS layer formed on the surface is believed to alleviate nZVI passivation, enabling a more sustained and efficient reactivity. Additionally, the BC support improved the dispersion and stability, which further facilitated electron transfer and promoted radical generation. When PS was introduced, the degradation efficiency of the nZVI/PS system increased by 38.61% compared with that of nZVI alone. Similar trends were observed when comparing nZVI@BC/PS with nZVI@BC and S-nZVI@BC/PS with S-nZVI@BC. PS-enhanced systems based on iron materials performed better than their non-PS counterparts. This was likely due to the dual function of iron-based materials: reducing crude oil and simultaneously activating PS to generate reactive radicals for oxidation, thereby improving the overall degradation performance.

#### 3.2.2 Optimization of S-nZVI@BC/PS system conditions

**FIGURE 4:**
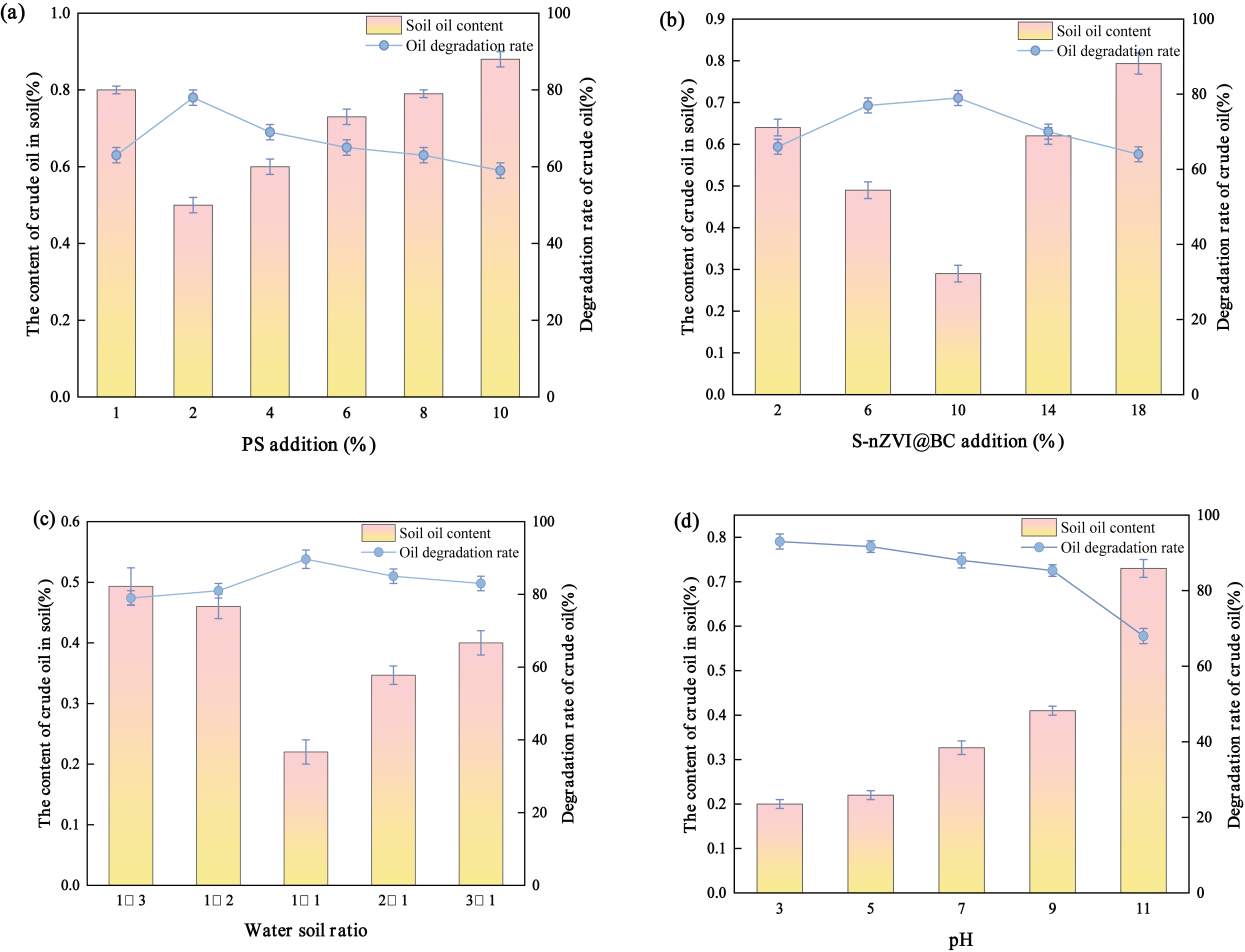
Effect of **(a)** PS addition, **(b)** S-nZVI@BC material addition, **(c)** different water-to-soil ratios, and **(d)** different pH values on the degradation rate of soil crude oil.

The degradation performance of the S-nZVI@BC/PS system varied with the PS concentration. A noticeable increase in crude oil removal efficiency was observed (Figure 4a). When the PS dosage increased from 1 to 2%, the degradation rate increased from 63.48 to 76.97%. This improvement was likely due to the increased number of contact sites between the PS and S-nZVI@BC as the PS concentration increased. More reactive sites promoted the generation of reactive radicals. Consequently, the probability of collisions between radicals and crude oil contaminants in the soil was enhanced, leading to an improved degradation efficiency. An increasing trend in removal efficiency with increasing PS dosage was observed, which is consistent with previous studies (Valentin et al., 2021; Xu et al., 2020). The maximum crude oil degradation efficiency was achieved at a PS dosage of 2%. However, as the PS concentration increased from 2 to 10%, the degradation rate decreased from 76.97 to 60.07%. This decrease was likely caused by a PS overdose. Excessive PS leads to radical quenching and reduces the effective concentration of SO_4_^–^∙ (Guo et al., 2020). In addition, the surplus SO_4_^–^∙ can self-react to form SO_4_^2–^ (Wang Q. et al., 2023). Overdosed PS (S_2_O_8_^2–^) may also react with SO_4_^–^∙, further generating SO_4_^2–^. These side reactions potentially consume the active sites on the surface of S-nZVI@BC, reducing its catalytic activity. Therefore, the PS dosage should be carefully controlled to avoid excessive degradation and ensure optimal degradation performance.

When the dosage of S-nZVI@BC increased from 2 to 10%, the crude oil degradation rate increased from 68.70 to 86.47% (Figure 4b). The highest degradation efficiency was observed at a 10% dosage. This enhancement was attributed to the increased number of active sites on the material surface, which released more Fe^2+^ ions (Wei et al., 2016). The elevated Fe^2+^ concentration promoted collisions with PS, facilitating the generation of SO_4_^–^∙ and enhancing the degradation of crude oil. However, when the dosage was increased from 10 to 18%, the degradation rate decreased from 86.47 to 64.49%. This decline was likely caused by excess Fe^2+^ produced at higher dosages. The surplus Fe^2+^ consumed SO_4_^–^∙ through side reactions, forming SO_4_^–^∙ and suppressing the degradation efficiency of the system.

The crude oil degradation rate increased from 77.56 to 90.14% as the soil-to-water ratio increased from 1:3 to 1:1 (Figure 4c). The highest efficiency was achieved at a 1:1 ratio. This improvement was likely due to the transition of the system from a solid phase to more fluid conditions. This enhanced mobility facilitated the dissolution of crude oil in the aqueous phase. This promoted adsorption onto the BC surface and increased the contact between PS and pollutants. Consequently, more collisions occurred between SO_4_^–^∙ and crude oil compounds. However, when the ratio increased from 1:1 to 3:1, the degradation efficiency dropped from 90.14 to 82.44%. This decline was likely caused by the dilution of PS, which reduced the SO_4_^–^∙ concentration. Additionally, excess water favored side reactions between SO_4_^–^∙ and water, generating ∙OH with lower selectivity and shorter lifetime. Together, these effects diminish the degradation performance of the system.

The degradation rates corresponding to the five pH values were 90.93, 90.14, 84.64, 80.97, and 66.99% (Figure 4d). Acidic conditions favor the activation of PS by iron-based materials (Zhang et al., 2022). Specifically, the degradation rate decreased by 6.29% when the pH was increased from 3 to 7. This was likely because SO_4_^–^ served as the dominant radical under acidic conditions. Fe^2+^ was released from S-nZVI@BC and continuously activated PS to generate SO_4_^–^. Additionally, PS reacted with H^+^ to produce SO_4_^–^, enhancing the degradation performance of the system. When the pH was increased from 7 to 11, the degradation rate decreased by 17.65%, from 84.64 to 66.99%. This reduction was likely due to the reaction between excessive ∙OH and SO_4_^–^, which produced SO_4_^2–^ and reduced the SO_4_^–^ concentration. Moreover, ∙OH exhibited lower selectivity and a shorter lifetime, leading to weaker degradation efficiency compared to SO_4_^–^. Under alkaline conditions, a large amount of ∙OH^–^ reacted with Fe^2+^ to form hydroxide precipitates. Thus, the generation of radicals in the nZVI@BC/PS system was reduced. These precipitates were deposited on the active sites of nZVI@BC, hindering PS activation and decreasing the degradation efficiency. Therefore, acidic conditions favored the remediation of crude oil-contaminated soil by the nZVI@BC/PS system, whereas alkaline conditions suppressed its degradation performance.

#### 3.2.3 Effects of common inorganic salts in soil

**FIGURE 5:**
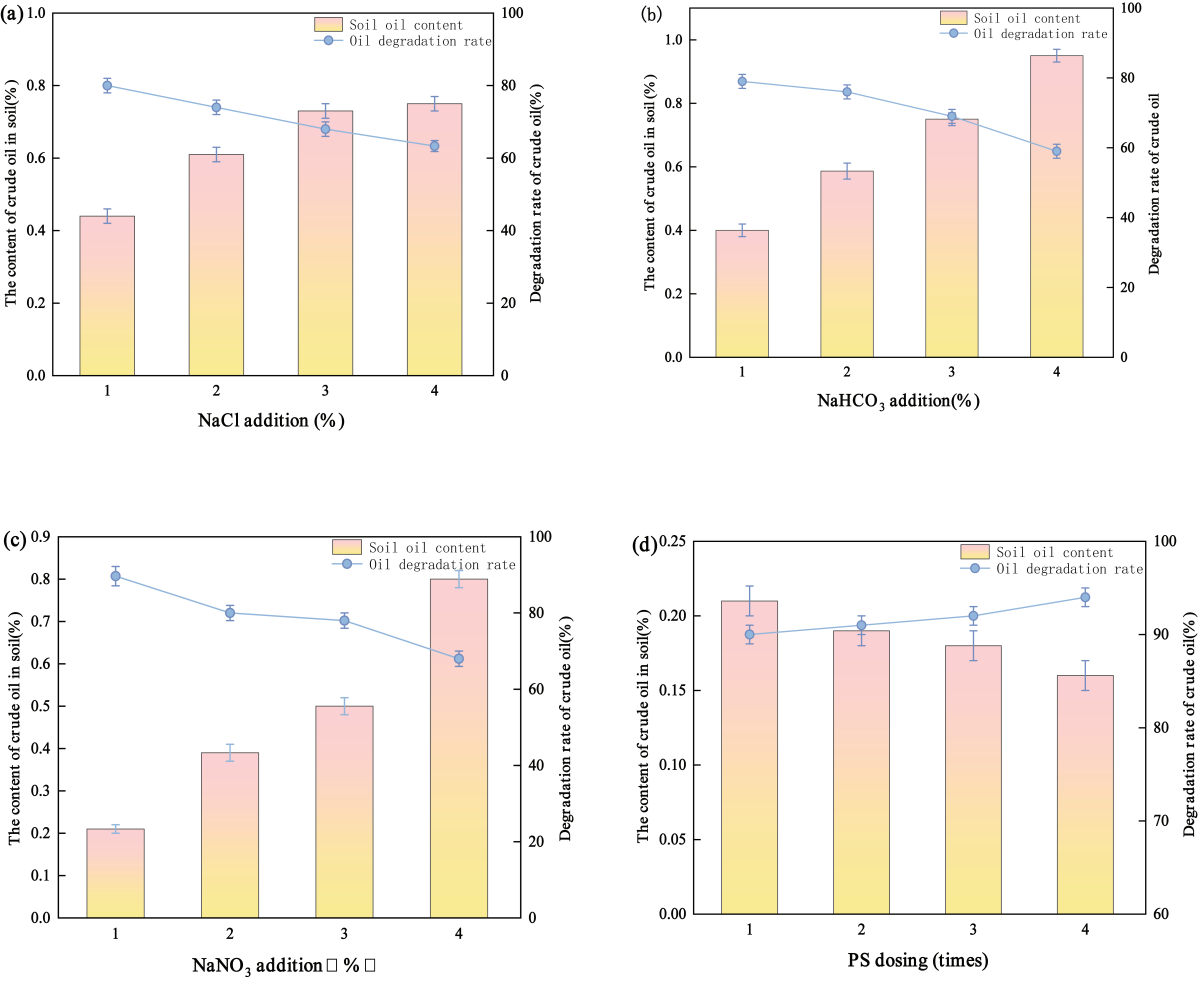
Effects of different **(a)** chloride ion, **(b)** bicarbonate ion, and **(c)** nitrate ion concentrations, and **(d)** the number of times sodium PS was added on the degradation rate of crude oil in soil.

Natural soils contain abundant inorganic anions. These species may participate in the radical-induced oxidation reactions and potentially influence the degradation efficiency of the S-nZVI@BC/PS system. Figure 5a shows the effect of NaCl addition on the crude oil removal. When NaCl was added at concentrations of 1, 2, 3, and 4%, the degradation efficiencies were 79.83, 72.28, 67.28, and 65.92%, respectively. Compared with the control group, the degradation rates decreased by 10.31, 17.86, 22.86, and 24.22%, respectively. The decline may have been caused by Cl^–^ interfering with the system. High Cl^–^ concentrations possibly led to the formation of iron–chloride complexes on the S-nZVI@BC surface, passivating active sites and hindering electron transfer. In addition, Cl^–^ likely scavenged SO_4_^–^ and ∙OH, reducing the availability of reactive species. These results indicated that Cl^–^ exerted an inhibitory effect on crude oil degradation in the S-nZVI@BC/PS system.

As shown in Figure 5b, the crude oil degradation efficiencies with 1, 2, 3, and 4% NaHCO_3_ are 81.93, 73.09, 65.83, and 57.65%, respectively. This inhibition was likely due to the radical scavenging effect of HCO_3_^–^. It reacted with SO_4_^–^ and ∙OH, forming SO_4_^2–^ and ∙OH^–^, thereby reducing reactive radical concentrations (Liu et al., 2019; Chen T. et al., 2019). Additionally, excessive HCO_3_^–^ increased system pH, shifting the environment to alkaline. Under such conditions, Fe^2+^ reacted with ∙OH^–^ and CO_3_^2–^ to form Fe(CO_3_)(OH)^–^, reducing Fe^2+^ activation capacity toward PS. Complexes formed between Fe^2+^ and ∙OH^–^ also suppressed PS activation. Under acidic conditions, HCO_3_^–^ reacted with H^+^ to produce CO_2_ and H_2_O. The resulting CO_3_^2–^ reacted with SO_4_^–^ and ∙OH to form weaker CO_3_^–^, further impairing degradation performance. Overall, bicarbonate exerts a strong inhibitory effect on crude oil degradation in the S-nZVI@BC/PS system.

The degradation efficiencies under 1, 2, 3, and 4% NaNO_3_ addition were 89.88, 82.12, 77.42, and 64.45%, respectively (Figure 5c). An overall decreasing trend in crude oil removal was observed. This reduction was likely caused by NO_3_^–^ interference. NO_3_^–^ is activated by S-nZVI@BC/PS to generate NO_3_, which is adsorbed onto the S-nZVI@BC surface. This adsorption likely led to passivation of the active sites. Additionally, the redox potential of NO_3_ is significantly lower than that of SO_4_^–^∙, resulting in a weaker oxidative capacity toward the target pollutants (Sbardella et al., 2019). NO_3_^–^ accelerates the aging of nZVI (Sun et al., 2016). Excess NO_3_^–^ may oxidize nZVI, causing its depletion. Furthermore, NO_3_^–^ competed for surface active sites on S-nZVI@BC, inhibiting PS activation. Thus, nitrate exerts a suppressive effect on the degradation efficiency of the S-nZVI@BC/PS system.

#### 3.2.4 Dosing frequency of PS

Nam et al. (2001) found that under the same total oxidant dosage, increasing the dosing frequency enhanced free radical utilization, thereby improving the pollutant removal efficiency. In this study, the S-nZVI@BC/PS system was evaluated under different persulfate (PS) dosing frequencies (1, 2, 3, and 4 additions). In all cases, a PS solution with a concentration of 0.84 mol/L was applied, and the total dosage was fixed at 2% (PS/Soil). The results showed that the petroleum degradation efficiencies for 1, 2, 3, and 4 PS additions were 90.14, 91.64, 91.99, and 93.37%, respectively (Figure 5d). As the dosing frequency increased, the degradation efficiency improved. The highest degradation rate was observed with four PS additions, which was 3.23% higher than that achieved with a single dose. This improvement was likely due to more effective utilization of SO_4_^–^ generated by S-nZVI@BC when PS was added in stages. Multiple dosing likely reduces the risk of radical quenching due to PS overdose. These findings were consistent with those reported by Lemaire et al. (2019).

### 3.3 Mechanism of degradation of crude oil contaminated soil by the S-nZVI@BC system

**FIGURE 6:**
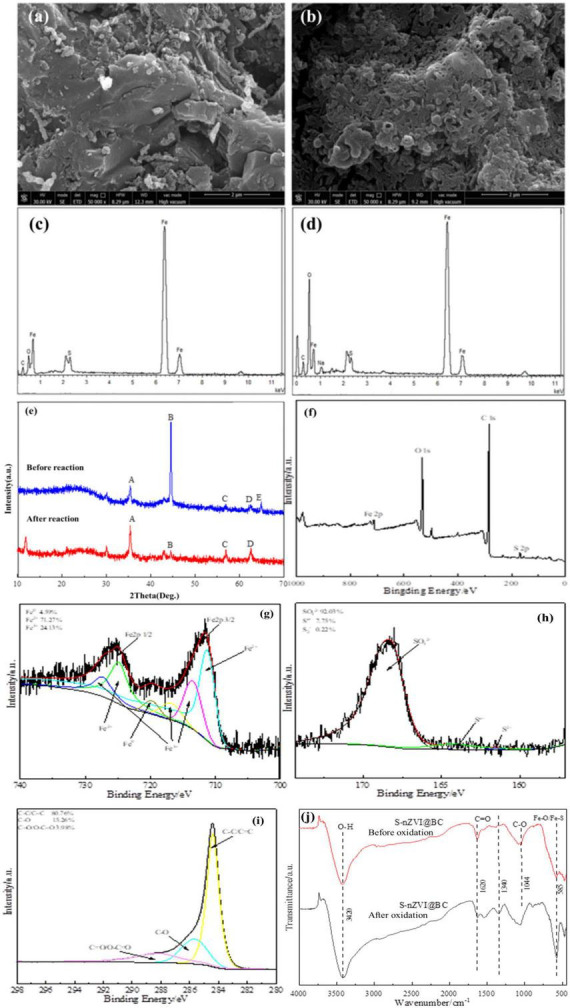
**(a)** S-nZVI @BC/PS system. **(a,c)** SEM images of S-nZVI@BC materials before oxidation, **(b,d)** SEM images of S-nZVI@BC materials after oxidation, **(e)** comparison of XRD patterns of S-nZVI@BC materials before and after oxidation, **(f–i)** XPS patterns of S-nZVI@BC materials after oxidation, and **(j)** comparison of FT-IR patterns of S-nZVI@BC materials before and after oxidation.

As shown in Figures 6a–d, the number of spherical particles on the surface of the oxidized S-nZVI@BC decreases (Ghauch et al., 2009). The surface became rougher, more porous, and more densely covered with aggregated structures. Morphological changes were also observed. These changes are likely caused by electron transfer during PS activation, in which Fe^2+^ is oxidized to Fe^3+^ cations by H_2_O. EDS analysis showed an increase in the oxygen content after oxidation, indicating that S-nZVI@BC underwent oxidative reactions during PS activation. As shown in Figure 6e, the diffraction peaks at 2θ = 35.38° (A) and 2θ = 56.72° (C) become more intense after oxidation, indicating the formation of Fe_2_O_3_ or Fe_3_O_4_. The peak at 2θ = 44.56° (B) significantly weakened, suggesting a notable reduction in zero-valent iron content. This is likely due to electron transfer from Fe^0^ to PS during activation, resulting in the formation of Fe^2+^ and Fe^3+^. Fe^3+^ further forms iron oxides. The enhanced peak at 2θ = 62.21° (D) indicates the generation of Fe(OH)_3_, which may have been deposited on the surface as Fe^3+^ reacted with H_2_O after PS activation. The disappearance of the peak at 2θ = 64.86° (E) suggests that the FeS shell on the S-nZVI@BC surface was involved in the reaction. These observations are consistent with the expected results.

As shown in the XPS spectra of the oxidized S-nZVI@BC (Figures 6f–i), the contents of Fe^0^ and Fe^3+^ decrease after the reaction, whereas the proportion of Fe^2+^ increases. Specifically, Fe^0^ and Fe^3+^ decreased to 4.59 and 24.13%, respectively, whereas Fe^2+^ increased to 71.27%. This indicates that Fe^0^ participated in the reaction and that portions of Fe^0^ and Fe^3+^ were converted to Fe^2+^ during PS activation. The contents of S_*n*_^–^ and S^2–^ declined from 17.66 and 8.92% to 7.75 and 0.22%, respectively, whereas SO_4_^2–^ increased from 73.42 to 92.03%. This suggested that S_*n*_^–^ and S^2–^ were oxidized to SO_4_^2–^, likely due to exposure to air and electron transfer during PS activation. The presence of S_*n*_^–^and S^2–^ may have further promoted the reduction of Fe^3+^ to Fe^2+^ (Mandal et al., 2019). After the reaction, the C-C/C = C content increased from 63.33 to 80.76%, whereas the proportions of C-O and C = O/O-C = O decreased from 29.64 and 7.03% to 15.26 and 3.98%, respectively. This indicated that the oxygen-containing functional groups on the BC surface participated in electron transfer during the reaction. These groups likely contribute to crude oil adsorption and PS activation. Moreover, BC acts as an effective conductor, promoting electron cycling between the Fe^2+^ and Fe^3+^.

As shown in Figure 6j, the FT-IR spectra of the S-nZVI@BC composite before and after oxidation exhibit several changes. The O-H stretching vibration peak at 3,420 cm^–1^ decreased slightly after the reaction, likely because of the reduced adsorbed moisture on the material surface. The C = O stretching peak at 1,620 cm^–1^ also weakened, and the C-O stretching peak at 1,044 cm^–1^ exhibited a noticeable shift, suggesting the involvement of these functional groups in the reaction. A pronounced absorption peak appeared at 1,340 cm^–1^, corresponding to metal oxides. In addition, the Fe-O/Fe-S stretching vibration peak at 585 cm^–1^ intensified, indicating the formation of iron oxides during PS activation. These results are consistent with those of previous characterization analyses.

**FIGURE 7:**
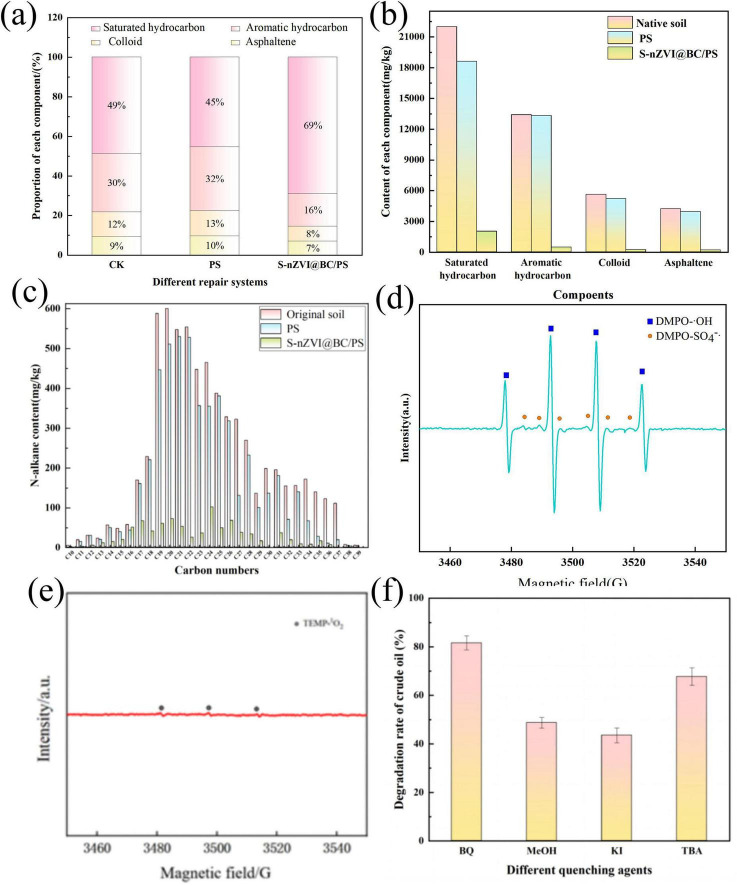
**(a)** S-nZVI@BC/PS system in Figure 6. **(a)** Comparison of the proportion of the four crude oil components before and after oxidation, **(b)** comparison of the content of the four crude oil components before and after oxidation, **(c)** Changes in the n-alkane (C10–C40) content in the soil before and after oxidation, **(d,e)** EPR images after oxidation, and **(f)** effects of different quenchers on the degradation rate of soil crude oil.

Changes in the four crude oil fractions were compared among the untreated (control), PS-treated, and S-nZVI@BC/PS-treated soils (Figure 7a). In the original soil, the saturated hydrocarbon, aromatic, resin, and asphaltene contents were 49, 30, 12, and 9%, respectively. In the PS group, saturated hydrocarbons decreased by 4%, whereas aromatics, resins, and asphaltenes increased by 2, 3, and 1%, respectively. This was likely due to the activation of PS by natural organic matter in the soil, which partially degraded crude oil compounds. In the S-nZVI@BC/PS group, compared to the control, aromatics, resins, and asphaltenes decreased by 14, 2, and 4%, respectively, whereas saturated hydrocarbons increased by 20%. This increase was possibly attributed to the cleavage of complex and high-molecular-weight components (e.g., aromatics, resins, and asphaltenes) by SO_4_^–^ generated during PS activation, leading to the formation of simpler saturated hydrocarbons. These results indicate that the S-nZVI@BC/PS system exhibited a higher degradation efficiency for aromatics, resins, and asphaltenes, whereas the removal of saturated hydrocarbons was comparatively lower. As shown in Figure 7b, the degradation efficiencies of saturated hydrocarbons, aromatics, resins, and asphaltenes in the PS-treated soil are 15.00, 0.70, 7.00, and 7.00%, respectively, compared with those in the untreated soil. In contrast, the S-nZVI@BC/PS-treated soil exhibited significantly higher degradation efficiencies of 91.00, 96.00, 96.00, and 95.00% for the same four fractions. These results indicate that the S-nZVI@BC/PS system effectively degraded all major crude oil fractions in the contaminated soil.

In untreated soil, the predominant n-alkanes ranged from C_11_ to C_37_. After the addition of PS, the concentrations of C_10_–C_21_ and C_22_–C_37_ decreased by 3–142 mg/kg and 9–192 mg/kg, respectively (Figure 7c). This was likely due to the activation of PS by natural organic matter in the soil, which led to the cleavage of long-chain n-alkanes into shorter fragments. In the S-nZVI@BC/PS-treated group, the C_10_–C_21_ and C_22_–C_37_ levels decreased by 4–529 and 91–528 mg/kg, respectively. These results indicated that the PS and S-nZVI@BC/PS systems degraded n-alkanes, with the S-nZVI@BC/PS system exhibiting superior degradation performance.

DMPO and TEMP were used to capture SO_4_^–^, ∙OH, and ^1^O_2_ generated in the S-nZVI@BC/PS system after 20 min of reaction. The species were identified using EPR (Figures 7d,e). A quartet signal with an intensity ratio of 1:2:2:1 was observed, which is consistent with DMPO-OH, confirming the presence of ∙OH (Ye et al., 2009). A sextet signal (1:1:1:1:1:1), corresponding to DMPO-SO_4_^–^, was also detected, indicating the formation of SO_4_^–^ (Li et al., 2023). The signal intensity of DMPO-SO_4_^–^ appeared weaker than that of DMPO-OH. However, this did not imply that ∙OH dominated the system, as SO_4_^–^ could be transformed into ∙OH during the reaction. Furthermore, the reaction rate constant between DMPO and ∙OH is higher than that between DMPO and SO_4_^–^ (Fang et al., 2021), making DMPO-OH easier to detect. Additionally, a triplet signal with an intensity ratio of 1:1:1, attributed to TEMP-^1^O_2_, was observed, confirming the presence of singlet oxygen in the system alongside SO_4_^–^andOH.

As previously described, the degradation efficiency of S-nZVI@BC/PS for crude oil in the soil was 93.37% in the absence of radical scavengers (blank control). Upon the addition of MeOH, TBA, PBQ, and KI, the degradation efficiencies decreased to 58.13, 77.74, 83.54, and 49.58%, respectively (Figure 7f). Upon the addition of MeOH and TBA, the degradation efficiency of S-nZVI@BC/PS for crude oil decreased by 35.21 and 15.60%, respectively. This indicated that SO_4_^–^ and ∙OH played dominant roles in the degradation process, with SO_4_^–^ contributing more significantly. In contrast, the addition of PBQ resulted in only a 9.80% reduction in the degradation efficiency, suggesting a minor role for superoxide radicals. This was likely due to the presence of CO_3_^2–^ in the soil, which effectively quenched O_2_^–^ (Cheng et al., 2017). The most pronounced inhibition was observed with KI, which caused a 43.76% decrease in degradation efficiency, indicating that the primary reactive sites were located on the surface of S-nZVI@BC.

### 3.4 Changes in soil microbial community structure and soil toxicity

Coverage values in Table 1 were ≥ 98% for all groups, indicating adequate representativeness of the soil microbial communities. The contaminated soil had ACE and Chao indices of 1155.54 and 1093.38, respectively. Relative to the CK group, ACE and Chao values decreased to varying degrees in the other nine treatments, indicating reduced microbial richness. The largest decline was observed in the PS group: ACE decreased by 215.49 and Chao decreased by 186.66. The PS-amended treatments also showed decreases in both indices, suggesting that PS addition inhibited a portion of the microbial community.

**TABLE 1:** Data Sheet for alpha diversity analysis of soil samples.

Samples	Systems	Ace	Chao	Coverage	Shannon	Simpson
Z1-1	nZVI	949.65	910.29	1.00	3.51	0.1
Z1-2	nZVI@BC	998.22	945.08	1.00	3.91	0.06
Z1-3	S-nZVI@BC	1086.38	1082.1	1.00	4.02	0.06
Z2-1	nZVI/PS	1107.18	1064.22	1.00	3.78	0.07
Z2-2	nZVI@BC/PS	1060.96	1010.97	1.00	4.2	0.05
Z2-3	S-nZVI@BC/PS	1139.51	1082.1	1.00	3.73	0.05
Z2-4	BC/PS	1002.01	945.08	1.00	3.76	0.07
Z3-1	PS	940.05	906.72	1.00	3.03	0.27
Z3-2	CK	1155.54	1093.38	1.00	4.45	0.03
Z3-3	BC	1054.47	1023.99	1.00	3.93	0.05

Compared to Z3-2, the ACE and Chao indices of Z1-1 decreased by 205.89 and 245.26. This suggests a mild toxicity to the microbial community. Compared to Z1-1, Z1-2, and Z1-3, ACE increased by 48.57 and 136.74 and Chao increases by 34.80 and 171.81, respectively. These gains were likely due to the BC support, which reduced the contact area between nZVI and the microbes, thus mitigating nZVI toxicity (Hou et al., 2021). The smallest declines in ACE and Chao were observed in Z2-3, indicating that the sulfidation of nZVI combined with BC loading reduced the toxicity associated with nZVI and PS.

Relative to Z1-1, the Shannon indices of nZVI@BC and S-nZVI@BC/PS increased by 0.04 and 0.51, whereas the Simpson indices decreased by 0.04 and 0.03, respectively. After PS was added to these systems, Shannon increased by 0.27 and 0.69, and Simpson decreased by 0.03 and 0.05, respectively. These results indicated that BC loading and nZVI modification reduced nZVI toxicity. Among the 10 treatments, the PS group exerted the most pronounced effect. nZVI is toxic to microbes; however, sulfidation and BC support effectively mitigate the adverse effects of PS and nZVI. The S-nZVI@BC/PS system had the smallest impact on the soil microbial diversity.

**FIGURE 8:**
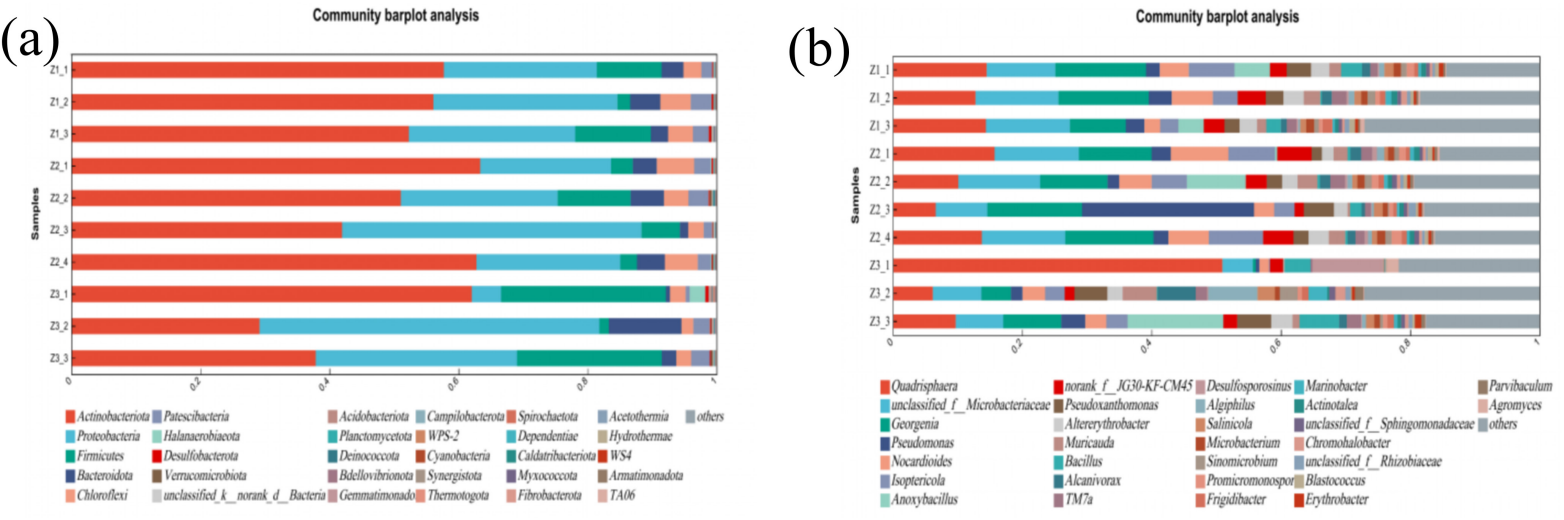
Soil microbial community structure **(a)** at the phylum and **(b)** genus levels under different treatments.

Studies have shown that Proteobacteria and Firmicutes contain many crude oil-degrading taxa and are important for soil remediation (Jurado et al., 2014; Wu et al., 2019). In Z3-2, Proteobacteria accounted for 52.67%. After treatment, the relative abundance of Proteobacteria changed by varying degrees (Figure 8a). The largest decrease occurred in Z3-1, indicating that the strong oxidizing power of PS likely suppressed the activity of Proteobacteria. In contrast, Z2-3 showed only a 6.27% decrease, suggesting a comparatively low toxicity of the S-nZVI@BC/PS system to Proteobacteria. In Z3-2, Firmicutes had the lowest baseline abundance. Following treatment, the abundance of Firmicutes increased to different extents. The greatest increase was observed in Z3-1, implying that PS may have promotes the growth of Firmicutes. In Z2-3 (S-nZVI@BC/PS), Firmicutes increased by 4.51%, indicating that the soils remediated by this system supported Firmicutes.

At the genus level, thirty genera and one unclassified group denoted as “others” were detected after treatment (Figure 8b). Relative to Z3-2, the abundance of Nocardioides markedly increased in Z1-2, Z2-1, and Z2-4, where it emerged as the dominant genus. This genus was reported by Ma et al. (2023) to degrade recalcitrant pollutants, including aromatic compounds, hydrocarbons, haloalkanes, N-heterocycles, and polyester polymers. Compared with the contaminated soil, Bacillus abundance increased in Z1-1, Z2-3, Z3-1, and Z3-3. As noted by Patowary et al. (2016) and Zhang et al. (2021), Bacillus can degrade total crude oil and PAHs, and tolerate adverse soil conditions. Pseudomonas is a versatile degrader. According to Leahy and Colwell (1990), it removes saturated hydrocarbons, mono- and polycyclic aromatics, and resinous fractions. Pseudoxanthomonas was relatively abundant in Z2-3, Z3-2, and Z3-3 and acted as a key crude oil-degrading genus. Kim et al., 2008 reported BTEX degradation by this genus, and Wei et al. (2021) documented the removal of PAHs and phenol. Relative to contaminated soil, Pseudomonas increased in Z1-2, Z2-1, and Z2-3. The largest increase occurred in Z2-3, where the relative abundance increased by 24%.

*Elymus dahuricus* seeds germinated well in uncontaminated soil, with a germination rate of 95.78% (Table 2). In crude oil-contaminated soil, the germination rate dropped to 24.39%, and root and shoot lengths were reduced to 3.37 and 3.86 mm, respectively. Relative to the uncontaminated soil, germination decreased by 75.61% and root and shoot lengths declined by 15.84 and 13.49 mm. These results indicate marked phytotoxicity that impedes seed germination and normal plant growth. After remediation with the S-nZVI@BC/PS system, germination, root length, and shoot length increased by 68.79%, 9.05, and 8.78 mm compared to the contaminated soil. After S-nZVI@BC/PS treatment, the oil content in the contaminated soil was 0.15%, and soil toxicity was significantly reduced.

**TABLE 2:** Wheat seed germination rate and root length and sprout length.

Soil type	Seed germination rate (%)	Root length (mm)	Shoot length (mm)
Uncontaminated soil	95.78 (100)	19.21 (100)	17.35 (100)
Contaminated soil	23.36 (24.39)	3.37 (17.54)	3.86 (22.24)
S-nZVI@BC/PS-remediated soil	89.25 (93.18)	12.42 (64.65)	12.64 (72.85)

## 4 Discussion

**FIGURE 9:**
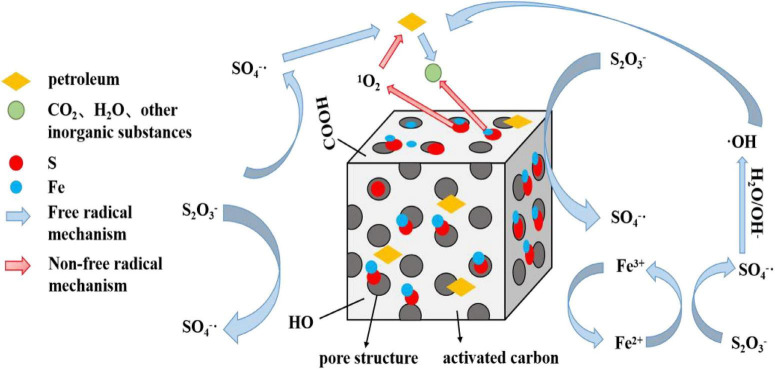
Mechanism of soil crude oil degradation by the S-nZVI@BC/PS system.

Based on the degradation performance of the S-nZVI@BC/PS system and the compositional changes in residual crude oil, the enhanced oxidation efficiency can be attributed to a synergistic dual-pathway mechanism involving radical and non-radical processes (Figure 9). The superior catalytic activity originates not simply from reactive iron species, but from the structural and electronic coupling between S-nZVI and BC, which regulates PS activation. BC functions as both a dispersive support and a conductive matrix. Its porous structure adsorbs crude oil components and PS, increasing local reactant concentrations and facilitating interfacial reactions. Meanwhile, the conductive carbon framework promotes electron transfer, which is essential for sustaining iron redox cycling. The FeSX shell on S-nZVI further enhances electron transfer efficiency while mitigating rapid passivation. The dominant pathway is radical-mediated oxidation. Fe^2+^ generated from Fe^0^ activates PS to produce SO_4_^–^, which rapidly oxidize aliphatic and aromatic hydrocarbons. As Fe^3+^ accumulates, electron transfer from Fe^0^ regenerates Fe^2+^, maintaining continuous PS activation. The conductive BC matrix accelerates this Fe^2+^/Fe^3+^ redox cycling, thereby sustaining radical production. These results indicate that enhanced degradation efficiency is governed by improved electron transfer kinetics rather than iron content alone. A non-radical pathway also contributes to degradation. Oxygen-containing functional groups and defect sites on BC can activate PS to generate ^1^O_2_, which selectively oxidizes hydrocarbons. However, partial coverage of BC surface functional groups by S-nZVI reduces their accessibility, limiting the relative contribution of BC-mediated activation. Thus, while BC enhances electron mobility and iron dispersion, its intrinsic catalytic role in PS activation is partly suppressed.

Overall, radical and non-radical pathways likely coexist and complement each other. Radical oxidation dominates during early stages due to rapid Fe^2+^-mediated activation, whereas BC-associated non-radical processes may contribute progressively as the reaction proceeds. The coupling of adsorption, electron transfer, and Fe redox cycling explains the sustained degradation performance and the observed compositional transformation of crude oil. This mechanistic framework highlights the importance of structural synergy in regulating PS activation and provides a more convincing interpretation of the system’s catalytic behavior.

In recent years, BC@nZVI and S-nZVI/S-ZVI activated persulfate systems have attracted considerable attention for the remediation of organic-contaminated soils (Supplementary Table S2). Zeng et al. (2022) demonstrated that using biochar as a support significantly improves the dispersion and electron transfer capacity of nZVI, thereby enhancing persulfate activation efficiency and achieving the effective removal of aromatic hydrocarbons under pilot-scale field conditions. Meanwhile, Chung et al. (2024) reported that sulfidation forms an FeS_*x*_ shell on the iron surface, which enhances electron donation and suppresses surface passivation, thus promoting sustained persulfate activation. However, most previous studies have focused on individual target pollutants such as naphthalene, phenanthrene, anthracene, or phenol (Li et al., 2025; Zhou et al., 2023b), with limited attention paid to crude oil-contaminated soils characterized by complex compositions and aging effects. In this study, the proposed S-nZVI@BC/PS system achieved efficient persulfate activation and the synergistic removal of multiple components in complex crude oil-contaminated soil, thereby extending the applicability of these systems toward the practical remediation of real-world petroleum-contaminated sites. Despite the promising degradation performance observed, several limitations should be acknowledged. First, ecological evaluation was limited to seed germination tests, which mainly reflect acute phytotoxicity and cannot fully represent long-term plant growth or agricultural safety. Second, the experiments were performed in laboratory-scale batch systems under controlled conditions, which may not fully reflect the complexity of field environments. In addition, the study focused on standardized soil particle size fractions for comparative purposes; however, hydrocarbon distribution and removal efficiency may vary among different particle size ranges. Future studies should further conduct field-scale validation and systematically evaluate soil microbial community responses, ecotoxicological impacts, the fate and transformation of iron/sulfur species, and material recoverability, in order to facilitate the transition of the S-nZVI@BC/PS system from laboratory investigations to practical field-scale remediation applications.

## 5 Conclusion

This study systematically investigated the application of S-nZVI@BC as an efficient PS activator for the remediation of crude oil-contaminated soil, with particular emphasis on structural optimization, degradation performance, and ecological implications. The results demonstrate that the catalytic efficiency of the system is strongly dependent on the S/Fe molar ratio, BC loading rate, and particle size. Under optimized conditions (S/Fe = 1:10, 25% BC loading, 850–425 μm particle size), a maximum degradation efficiency of 93.37% was achieved, indicating the high effectiveness of the composite material in activating PS for petroleum hydrocarbon removal. Mechanistic investigations revealed that the enhanced degradation performance originated from the structural synergy between sulfidated nanoscale zero-valent iron and biochar. The conductive BC matrix facilitated electron transfer and promoted Fe^2+^/Fe^3+^ redox cycling, while the FeSX-modified surface improved iron stability and PS activation efficiency. SO_4_^–^ and ∙OH were identified as the dominant reactive species, with SO_4_^–^ playing a primary role in hydrocarbon oxidation. This work highlights that improved electron transfer kinetics, rather than iron dosage alone, govern catalytic performance. In addition, although Cl^–^, HCO_3_^–^, and NO_3_^–^ exhibited inhibitory effects, the system maintained strong degradation capability, suggesting potential applicability under complex environmental conditions. Significant reductions were observed across all major crude oil fractions, including saturated hydrocarbons, aromatics, resins, and asphaltenes, as well as both light (C10-C21) and heavy (C22-C37) hydrocarbon ranges, indicating broad-spectrum removal capacity. Importantly, the remediated soil supported the growth of crude oil-degrading bacteria, and the germination rate of Elymus dahuricus increased substantially compared to untreated soil, approaching that of uncontaminated soil. These findings suggest that the S-nZVI@BC/PS system not only effectively removes petroleum hydrocarbons but also mitigates ecological toxicity and enhances soil biological functionality. Overall, this study demonstrates that coupling sulfidated zero-valent iron with biochar provides a structurally synergistic and mechanistically efficient strategy for PS activation in contaminated soils. The work offers new insights into the design of composite catalysts that integrate adsorption, electron transfer, and redox cycling, and provides a promising foundation for the development of sustainable field-scale remediation technologies.

## Data Availability

The original contributions presented in the study are included in the article/Supplementary material, further inquiries can be directed to the corresponding authors.
