# Supplementary material for: Synthesis of biochar-supported sulfidated nanoscale zero-valent iron and its application as a persulfate activator for remediation of crude oil-contaminated soil
Source: Front Microbiol. 2026 Apr 14;17:1783533. doi: 10.3389/fmicb.2026.1783533 (PMC13121344; doi:10.3389/fmicb.2026.1783533)
Supplement: Supplementary file 1 [file Table_1.docx]

**banSupplementary Material**

**Table S1 Soil contamination indicators**

| Indicator | Value |
| --- | --- |
| Oil content（%） | 2.26 |
| Water content（%） | 2.30 |
| Saturates（%） | 49.00 |
| Aromatics（%） | 30.00 |
| Resins（%） | 12.00 |
| Asphaltenes（%） | 9.00 |

**Table S2 Comparison of existing research**

| Reference | Pollutant / Matrix | Activator / System | Oxidant | Advantages | Limitations |
| --- | --- | --- | --- | --- | --- |
| Zeng et al., 2022 | Aromatic hydrocarbon-contaminated soil | nZVI/BC | PS | Demonstrated field-scale feasibility and long-term stability | No sulfidation interface introduced to further enhance anti-passivation and selectivity |
| Wang et al., 2025 | Naphthalene (NAP)-contaminated soil | BC@nZVI/PS | PS | Improved dispersion and electron transfer via biochar support | Focused on a single PAH; lacks validation in complex crude oil systems |
| Zhou et al., 2023 | Phenanthrene (PHE) in soil-related system | BC@nZVI | PS | Provided mechanistic insight into radical contribution and iron redox cycle | Still dependent on nZVI surface activity; susceptible to soil matrix scavenging and passivation |
| Wan et al., 2024 | Chloronitrobenzene (NCB)-contaminated soil | nZVI/BC | PDS | Engineering-oriented evaluation with practical implications | Limited discussion on long-term stability and influence of complex soil organic matter |
| Chung et al., 2024 | Phenol-contaminated soil (in situ treatment) | S-ZVI/PS | PS | Improved anti-passivation performance and sustained electron release | Lacked biochar support; dispersion and mass transfer may be limited |
| Li et al., 2025 | Anthracene (ANT)-contaminated soil | BC-PAA@nZVI | PS | Enhanced dispersion and surface functionality | No sulfidation modification to further enhance resistance to passivation |
| Review: 2021 | Organic contaminants (soil/water) | Modified nZVI-activated PS | PS | Comprehensive mechanistic overview and modification strategies | Highlighted need for real soil validation, field-scale studies, and ecological risk assessment |


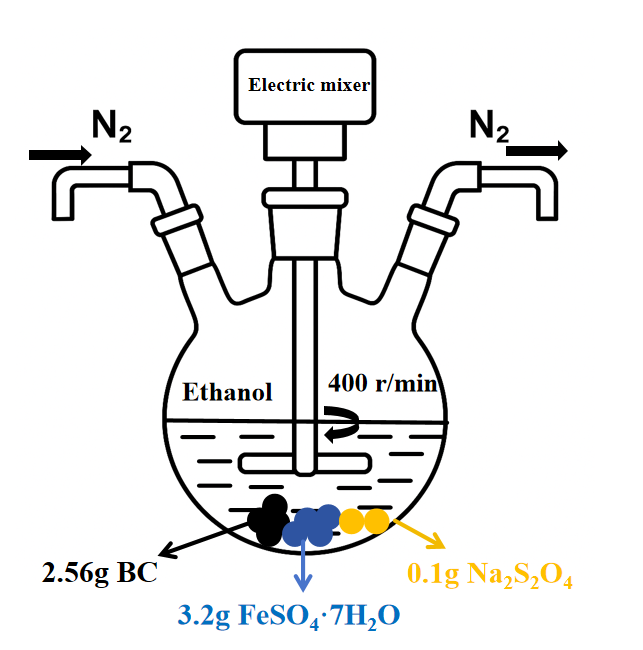


Fig.S1 Synthesis Flowchart

| 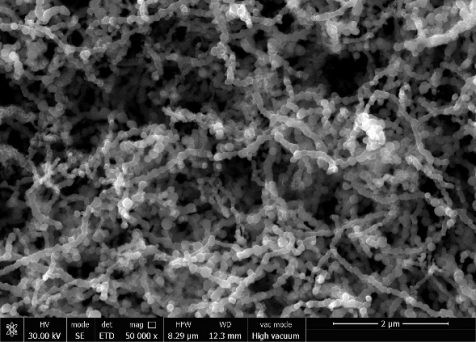 |
| --- |
| Fig. S2 SEM image of nZVI material |
| 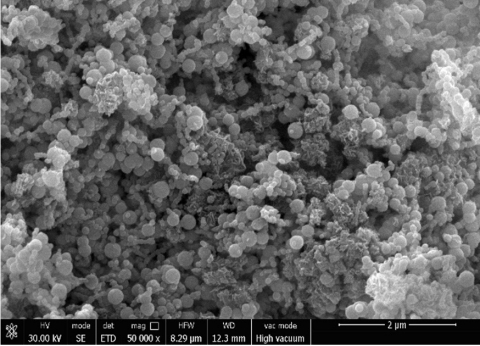 |
| Fig. S3 SEM image of S-nZVI material |

**Test S1**

Ferrous sulfate heptahydrate (FeSO_4_.7H_2_O) and dichloromethane (CH_2_Cl_2_) were obtained from Kermel Chemical Industry Co., Ltd. (Tianjin, China). Anhydrous ethanol (C_2_H_5_OH) and sodium borohydride (NaBH_4_) were purchased from Xilong Scientific Chemical Co., Ltd. (Shantou, China). Sodium hydrosulfite (Na_2_S_2_O_4_) and sodium PS (Na_2_S_2_O_8_) were purchased from Aladdin Biochemical Technology Co., Ltd. (Shanghai, China). Chloroform (CHCl_3_) and tetrachloroethylene (C_2_Cl_4_) were provided by Sinopharm Chemical Reagent Co., Ltd. (Beijing, China). n-Hexane (C_6_H_14_) was obtained from Acros Chemicals Co., Ltd. (Geel, Belgium) Anhydrous sodium sulfate (Na_2_SO_4_) was obtained from Sigma-Aldrich (MO, USA).
